# A human antibody against human endothelin receptor type A that exhibits antitumor potency

**DOI:** 10.1038/s12276-021-00678-9

**Published:** 2021-09-29

**Authors:** Man-Seok Ju, Hye-Mi Ahn, Seong-Gu Han, Sanghwan Ko, Jung-Hyun Na, Migyeong Jo, Chung Su Lim, Byoung Joon Ko, Yeon Gyu Yu, Won-Kyu Lee, Youn-Jae Kim, Sang Taek Jung

**Affiliations:** 1 Department of Biomedical Sciences, Graduate School, Korea University, Seoul, 02841 Republic of Korea; 2 Institute of Human Genetics, Korea University College of Medicine, Seoul, 02841 Republic of Korea; 3 Division of Translational Science, Research Institute, National Cancer Center, Goyang, Gyeonggi-do 10408 Republic of Korea; 4 Biopharmaceutical Chemistry Major, School of Applied Chemistry, Kookmin University, Seoul, 02707 Republic of Korea; 5 Department of Pharmaceutical Engineering, Sangji University, Wonju-si, Gangwon-do 26339 Republic of Korea; 6 Research Institute of Korean Medicine, Sangji University, Wonju-si, Gangwon-do 26339 Republic of Korea; 7 BK21 Graduate Program, Department of Biomedical Sciences, Korea University College of Medicine, Seoul, 02841 Republic of Korea; 8 New Drug Development Center, Osong Medical Innovation Foundation, Cheongju, Chungcheongbuk-do 28160 Republic of Korea; 9 School of Biopharmaceutical and Medical Sciences, Sungshin Women’s University, Seoul, 02844 Republic of Korea

**Keywords:** High-throughput screening, Antibody therapy, Antibody therapy

## Abstract

Endothelin receptor A (ET_A_), a class A G-protein-coupled receptor (GPCR), is involved in the progression and metastasis of colorectal, breast, lung, ovarian, and prostate cancer. We overexpressed and purified human endothelin receptor type A in *Escherichia coli* and reconstituted it with lipid and membrane scaffold proteins to prepare an ET_A_ nanodisc as a functional antigen with a structure similar to that of native GPCR. By screening a human naive immune single-chain variable fragment phage library constructed in-house, we successfully isolated a human anti-ET_A_ antibody (AG8) exhibiting high specificity for ET_A_ in the β-arrestin Tango assay and effective inhibitory activity against the ET-1-induced signaling cascade via ET_A_ using either a CHO-K1 cell line stably expressing human ET_A_ or HT-29 colorectal cancer cells, in which AG8 exhibited IC_50_ values of 56 and 51 nM, respectively. In addition, AG8 treatment repressed the transcription of inhibin βA and reduced the ET_A_-induced phosphorylation of protein kinase B and extracellular regulated kinase. Furthermore, tumor growth was effectively inhibited by AG8 in a colorectal cancer mouse xenograft model. The human anti-ET_A_ antibody isolated in this study could be used as a potential therapeutic for cancers, including colorectal cancer.

## Cancer: Human antibody shows therapeutic potential

A therapeutic antibody that targets a receptor involved in cancer progression shows significant anti-cancer effects in trials in mice. Endothelin receptor A (ET_A_) promotes the progression and metastasis of several cancers, and patients with high ET_A_ expression often have poor survival rates. Several small molecule drugs that target ET_A_ are currently undergoing trials. Now, Sang Taek Jung at the Korea University in Seoul, together with scientists across South Korea, have identified and isolated a human antibody that specifically binds to ET_A_. The team developed an antigen that mimics ET_A_, and identified and isolated the antibody it bound to. The antibody exhibited potent anti-tumor effects in cell cultures and trials in mice. Such therapeutic antibodies show higher affinity for their targets than other drugs, resulting in fewer side effects and higher efficacy.

## Introduction

G-protein-coupled receptors (GPCRs), the largest superfamily of membrane receptors in the human genome, transduce extracellular signals to the intracellular space through binding of their cognate ligands. Intracellular signaling events triggered by conformational changes in GPCRs and interactions with intracellular proteins regulate numerous cellular functions, such as growth, motility, and differentiation^1,2^. Because of the critical role of GPCRs in numerous biological functions, they are involved in the progression and prognosis of a variety of diseases and are the targets of ~35% of all commercialized drugs^3,4^.

Endothelin receptor type A (ET_A_) is a class A GPCR that belongs to the endothelin receptor family. It regulates blood vessel constriction, cell growth, and differentiation through several downstream signaling pathways activated by the binding of ligands such as ET-1, ET-2, and ET-3^5,6^. ET_A_, which undergoes a conformational change due to ligand binding, is involved in a variety of downstream signaling pathways through its interaction with G-protein alpha subunits (G_αs_, G_αq/11_, and G_αi_) in the intracellular space^7–9^. G_αs_ and G_αi_ control cell growth and motility in a manner dependent on the concentration of intracellular cyclic adenosine monophosphate (cAMP) produced by adenylyl cyclase, and G_αq/11_ regulates the intracellular Ca^2+^ concentration and cell proliferation through protein kinase C (PKC) and activation of phospholipase Cβ (PLCβ). Therefore, ET_A_ expression is closely related to the survival rates of patients with several types of cancers^10–12^, and endothelin receptor antagonists, including zibotentan, atrasentan, bosentan, macitentan, and ambrisentan, have been developed as drugs for treating cancer by inhibiting downstream ET_A_ signaling^13^. Currently, ET_A_ antagonists are being evaluated for their antitumor efficacy in a variety of preclinical and clinical trials for cancers such as melanoma, glioblastoma, prostate cancer, lung cancer, and colorectal cancer, which are closely related to the expression and activity of ET_A_^14^. However, all ET_A_ antagonists that have been evaluated for antitumor efficacy are small-molecule drugs.

Compared to small-molecule drugs, therapeutic antibodies have key advantages. First, they have extraordinarily high affinity and specificity for a target antigen, resulting in enhanced efficacy and reduced side effects. Second, they possess excellent Fc-mediated effector functions for clearance of target cells such as tumor cells. Third, they have prolonged circulating serum half-lives through pH-dependent binding of FcRn to the IgG Fc region^15,16^. However, developing therapeutic antibodies against GPCR targets is challenging because of the low expression levels of GPCR antigens on native cell membranes or in heterologous hosts, the difficulty of preparing a functional form of a GPCR antigen with a conformation similar to that of the complex seven transmembrane α-helical structure of native GPCRs, and the limited exposure of extracellular regions of GPCRs as a target for antibodies^17,18^. Due to these hurdles in developing anti-GPCR antibodies, only two therapeutic antibodies against GPCR antigens—erenumab (Aimovig^®^) and mogamulizumab (Poteligeo^®^), targeting the calcitonin gene-related peptide receptor and chemokine receptor 4, respectively—have been approved by the US FDA, in contrast to the clinical and marketing successes of a number of therapeutic antibodies targeting other types of antigens^17^.

In this study, we report the successful isolation of a human antibody antagonizing the functions of ET_A_ and the evaluation of its antitumor activity. ET_A_ nanodiscs were prepared by overexpressing ET_A_ in *E. coli* and reconstituting the detergent-solubilized form with lipids and membrane scaffold proteins (MSPs). Screening of an in-house-constructed human antibody phage display library against ET_A_ nanodiscs enabled us to isolate an antibody that binds specifically to ET_A_. The resulting human antibody regulating the downstream signaling of human ET_A_ showed potent antitumor effects in both in vitro tests and an in vivo xenograft mouse model. This study demonstrates that this antibody targeting human ET_A_ could be used to elucidate the functions of endothelin receptors and could be developed as a potential therapeutic agent for cancer.

## Materials and methods

### Reagents

All oligonucleotide primers and plasmids used in this study are described in Supplementary Tables 1 and 2. Restriction enzymes, Phusion^®^ High-Fidelity DNA polymerase, and T4 DNA ligase were purchased from New England Biolabs (Ipswich, MA, USA). Oligonucleotide primers and VCSM13 helper phage stock were obtained from Integrated DNA Technologies (Coralville, IA, USA) and Agilent Technologies (Santa Clara, CA, USA), respectively. 1-Palmitoyl-2-oleoylphosphatidylcholine (POPC), an anti-M13 antibody conjugated to horseradish peroxidase (HRP), and 1-Step^TM^ Ultra 3,3′,5,5′-tetramethylbenzidine (TMB) substrates were purchased from Avanti Polar Lipids (Alabaster, AL, USA), Bethyl Laboratories (Montgomery, TX, USA), and Thermo Fisher Scientific (Waltham, MA, USA), respectively. All other chemicals and reagents were purchased from Sigma-Aldrich (St. Louis, MO, USA) unless stated otherwise.

### Construction of plasmids

The mouse ET_A_ (mET_A_) gene (NCBI Gene ID: 13617) was synthesized by GenScript (Piscataway, NJ, USA). The pP9-mET_A_ plasmid was constructed by Gibson assembly^19^ of the mET_A_ DNA fragments amplified by polymerase chain reaction (PCR) using primers (MSJ#01 and MSJ#02) and the pP9 plasmid^20^ digested with the *Sma*I restriction enzyme. The gene encoding membrane scaffold protein (MSP-1), derived from the apolipoprotein A-I gene (NCBI Gene ID: 335), was assembled by PCR using primers (MSJ#03–MSJ#08) and was subcloned into pET28a(+) (Novagen, Burlington, MA, USA) at the *Nde*I/*Bam*HI restriction endonuclease sites to generate pET28-MSP-1. To construct plasmids encoding the heavy and light chains of full-length IgG for AG8, each VH and VL gene was PCR amplified using a phagemid (pEL3X-AG8) isolated from the phage library screen and the primer pairs MSJ#42/MSJ#44 for VH and MSJ#46/MSJ#48 for VL. Then, the DNA fragments encoding the IgG constant region (CH1-CH2-CH3) of trastuzumab, which were prepared by PCR amplification using a primer pair (MSJ#43/MSJ#45) and a template (pMAZ-IgH-GlycoT)^21^, were assembled with the VH DNA fragments using a primer pair (MSJ#42/MSJ#45). A primer pair (MSJ#46/MSJ#49) was used to assemble the DNA fragments for the VL gene, and the human C_k_ DNA fragments were amplified using primers (MSJ#47/MSJ#49) and a template (pMAZ-IgL-GlycoT)^21^. pMAZ-AG8H and pMAZ-AG8L were constructed by ligation of the resulting heavy and light-chain DNA of AG8 IgG, respectively, into the pMAZ-IgL-GlycoT plasmid at the *Bss*HII and *Xba*I sites.

### Expression and purification of human ET_A_, mouse ET_A_, and membrane scaffold protein-1

Human ET_A_ (hET_A_), mouse ET_A_ (mET_A_), and membrane scaffold protein-1 (MSP-1) proteins were expressed and purified as described in the literature^20,22^. *E. coli* BL21(DE3) harboring pP9-hET_A_^20^ or pP9-mET_A_ (for pP9-derived plasmids), or pET28a-MSP-1 (for pET28a-derived plasmids) was inoculated in Luria-Bertani (LB) medium supplemented with 100 μg/ml ampicillin (Millipore Sigma, Burlington, MA, USA) and 50 μg/ml kanamycin (Millipore Sigma, Burlington, MA, USA) and cultivated for 16 h at 37 °C and 250 rpm. Then, 100-fold dilutions of overnight-grown cells were inoculated in LB medium supplemented as needed with the same antibiotics and incubated at 37 °C until the absorbance of the culture broth at 600 nm (OD_600_) reached 0.6. After the addition of isopropyl-β-*D*-thiogalactopyranoside (IPTG) (0.5 mM for hET_A_ and mET_A_, 1 mM for MSP-1) and incubation under specific culture conditions (25 °C for 16 h for hET_A_ and mET_A_, 30 °C for 4 h for MSP-1) to induce protein expression, cells were harvested by centrifugation at 8000×*g* and disrupted using a microfluidizer (Microfluidics, Westwood, MA, USA). To prepare endothelin receptors (hET_A_ and mET_A_), the resulting lysates were centrifuged at 12,000×*g* for 20 min, and the supernatants were ultracentrifuged at 100,000×*g* for 1.5 h to recover the membrane fractions from the pellets. After the membrane fractions were dissolved in 0.5% sarkosyl and centrifuged at 30,000×*g* for 30 min to remove insoluble aggregates, the recovered supernatants were bound to Ni-NTA agarose (Qiagen, Germantown, MD, USA) equilibrated with Buffer A (25 mM Tris–HCl and 1 mM phenylmethylsulfonylfluoride (pH 7.8)). After the resin was washed with 20 column volumes (CV) of Buffer A supplemented with 20 mM imidazole, the resin-bound proteins were eluted using 5 CV of Buffer A supplemented with 300 mM imidazole. Then, the eluents were loaded onto a PD-10 desalting column (Cytiva, Marlborough, MA, USA) to remove excess imidazole, and the buffer was exchanged with 25 mM Tris–HCl (pH 7.8) containing 10% glycerol. The purified endothelin receptors (hET_A_ and mET_A_) were stored at −80 °C before use. To prepare MSP-1 proteins, cell lysates were centrifuged at 12,000×*g*, and the resulting supernatants were loaded onto a Ni-NTA column equilibrated with 10 ml of 50 mM Tris-Cl and 1% Triton X-100 (pH 7.4). After adding 10 ml of 50 mM Tris-Cl and 50 mM imidazole (pH 7.4) for washing and 10 ml of 50 mM Tris-Cl and 300 mM imidazole (pH 7.4) for elution, the eluent buffer was exchanged with 1 × phosphate-buffered saline (PBS, pH 7.4) containing 10% glycerol using a PD-10 desalting column.

### Preparation of reconstituted hET_A_ nanodiscs

Purified hET_A_ and MSP-1 were mixed with POPC dissolved in 100 mM sodium cholate at a hET_A_:MSP-1:POPC molar ratio of 1:30:60. After the addition of 200 mg/ml Bio-Beads^TM^ SM-2 (Bio-Rad, Hercules, CA, USA), the resuspended solution was incubated at 4 °C for 16 h with mixing by rotation at 100 rpm and centrifuged at 12,000×*g* for 5 min to remove detergents. Then, the supernatants were dialyzed in 1× PBS (pH 7.4) and concentrated using Amicon Ultra®4 spin columns (Merck Millipore; 30 kDa cutoff). The concentrated supernatants were loaded onto a Superdex 200 gel filtration chromatography column (Cytiva, Marlborough, MA, USA) for development in 35 ml of 1× PBS (pH 7.4), and the fractions showing both hET_A_ and MSP-1 protein bands in sodium dodecyl sulfate-polyacrylamide gel electrophoresis (SDS–PAGE) analysis were recovered.

### Construction of a human naive immune scFv library

VH and VL genes of human immunoglobulins, which were prepared from peripheral blood mononuclear cells (PBMCs) of anonymous donors as described in the literature^23^, were PCR amplified using 200 μM dNTPs, 1 μM mixed oligonucleotides (MSJ#07–MSJ#16 for VH and MSJ#17–MSJ#37 for VL), 2.5 units of Phusion^®^ High-Fidelity DNA polymerase, and 100 ng of cDNA as a template. Then, the VH and VL genes were assembled by PCR with two primers (MSJ#38/MSJ#39) to connect the resulting VH and VL genes with a flexible glycine–serine linker (GGGSSGGGGSGGGGSGGGGS), and the resulting PCR products encoding the single-chain variable fragments (scFvs) were digested with *Sfi*I and ligated into the pEL3X phagemid, which is a derivative of pComb3X^24^ with modified *Sfi*I sequences (GGCCCAGCCGGCC/GGCCTCGGGGGCC). Then, the ligation products were transformed into *E. coli* ER2738 (F´*proA*^*+*^*B*^*+*^ *lacI*^*q*^ *Δ(lacZ)M15 zzf::Tn*10(Tet^R^)*/fhuA2 glnV Δ(lac-proAB) thi-1 Δ(hsdS-mcrB)5*) to generate the human naive scFv antibody library.

### Preparation of phage particles from the scFv library

*E. coli* ER2738 cells harboring naïve immune scFv library plasmids were inoculated and grown for 1 h in 10 ml of Super Broth (SB) medium (Becton Dickinson Diagnostic Systems, Difco^TM^, USA) supplemented with 100 μg/ml carbenicillin. The culture broth was diluted 1:100 in 1 L of SB medium containing the same antibiotic and incubated at 37 °C with shaking at 250 rpm until the absorbance of the culture broth at 600 nm reached 0.8–1.0. Then, 1 ml of VCSM13 helper phage (1 × 10^12^ pfu) and 70 μg/ml kanamycin were added, and the infected cells were incubated for 16 h at 37 °C with shaking at 250 rpm to induce the production of scFv-displaying phage particles. The culture broth was centrifuged at 10,000×*g*, and the supernatants were mixed with polyethylene glycol (PEG)/NaCl solution containing 4% (w/v) PEG 8000 and 3% (w/v) NaCl. The pellets were resuspended in 1× PBS and 3% bovine serum albumin (pH 7.4), and the recovered phage particles were stored at 4 °C prior to use.

### Library panning and screening

In total, 50 μl of 4 μg/ml G_αi3_ protein purified as described previously^25^ was coated onto a 96-well plate (Corning, Corning, NY, USA) at 4 °C for 16 h. After extensive washing of the wells, 50 μl of hET_A_ reconstituted nanodiscs (4 μg/ml) was added, and the plate was incubated at room temperature for 2 h. Before loading the library phage particles into the wells of the plate immobilized with hET_A_ nanodiscs, a negative selection procedure was conducted. The library phage particles were incubated in wells immobilized with empty nanodiscs consisting of only MSP-1 and a lipid that did not contain hET_A_. Next, 50 μl of the resulting supernatants were added to the wells preimmobilized with hET_A_ nanodiscs. After the plate was washed with 1× PBS (pH 7.4), bound phage particles were eluted in 100 μl of glycine-HCl buffer (pH 2.2) and neutralized by the addition of 20 μl of 2 M Tris (pH 8.0). Then, 120 μl of the resulting neutralized, recovered phages and 1 ml of VCSM13 helper phage particles were added to infect *E. coli* ER2738, and the amplified phages were used for the next round of biopanning. The number of washing cycles was increased in each subsequent round of biopanning to enrich high-affinity binders. After five rounds of biopanning, *E. coli* ER2738 cells were infected with eluted phages, and 400 individual clones were cultured in 1 ml of SB medium at 37 °C with shaking at 250 rpm until the OD_600_ reached 0.6. Then, 50 μl of VCSM13 helper phages and 70 μg/ml kanamycin were added to the infected *E. coli* ER2738 cells. After overnight cultivation, the supernatant was used for phage enzyme-linked immunosorbent assay (ELISA).

### Phage ELISA

To isolate phage particles displaying specific anti-hET_A_ antibodies, 50 μl of 4 μg/ml purified human G_αi3_ protein diluted in 0.05 M Na_2_CO_3_ (pH 9.6) was added to each well of a 96-well plate (Corning, Corning, NY, USA) and incubated at 4 °C for 16 h. After blocking with 150 μl of 4% skim milk in 1× PBS (pH 7.4) and washing four times with 150 μl of PBS (pH 7.4) containing 0.02% n-dodecyl-β-d-maltoside (DDM), 50 μl of 5 μg/ml hET_A_ reconstituted nanodiscs was added to each well of the plate. Then, the plate was incubated at 25 °C for 1 h, washed with 150 μl of 1× PBS (pH 7.4) containing 0.02% DDM, and treated with 50 μl of rescued phage particles displaying scFvs. After incubating at 25 °C for 1 h and washing four times, 50 μl of anti-M13-HRP conjugates diluted 4000-fold in 1× PBS (pH 7.4) containing 0.02% DDM was added to the plate. After incubation for 1 h at 25 °C and four washes in 150 μl of 1× PBS containing 0.02% DDM (pH 7.4), 50 μl of 1-Step™ Ultra TMB was added to each well, and the plate was incubated for 20 min to develop the signal. After quenching the signal by the addition of 50 μl of 4 N H_2_SO_4_, the ELISA-binding signal was detected by measuring the absorbance at 450 nm in an Epoch plate reader (BioTek, Winooski, VT, USA).

### Luciferase assay

A luciferase assay was performed using a dual-luciferase reporter assay system (Promega, Madison, WA, USA) according to the manufacturer’s instructions. Poly-l-lysine was coated onto 96-well plates (Corning, Corning, NY, USA) by incubation at 37 °C for 1 h, and cells were then seeded at a density of 5 × 10^3^ cells/well. The luciferase reporter plasmids were cotransfected with the control plasmid encoding Renilla luciferase into the cells in the plate, and AG8 phage supernatants were added after 24 h. Then, a mixture of dye reagent was added after 48 h, and luciferase activity was measured using a VICTOR Light luminometer (PerkinElmer, Inc., Waltham, MA, USA). The transfection efficiency was evaluated by normalization to Renilla luciferase activity as a control.

### Mammalian cell culture

CHO-K1 cells expressing human ET_A_ were maintained as monolayer cultures on 100-mm cell culture dishes in Ham’s F12 medium supplemented with 10% fetal bovine serum (FBS) and 1 × antibiotic–antimycotic solution at 37 °C in a humidified atmosphere containing 5% CO_2_. The established human colorectal cancer cell lines HT-29 and HCT-116 were purchased from the Korean Cell Line Bank (Seoul, Korea) and maintained in HyClone RPMI-1640 medium (Cytiva, Marlborough, MA, USA) supplemented with 10% HyClone FBS (Cytiva, Marlborough, MA, USA), 1% penicillin-streptomycin, and 1% sodium pyruvate at 37 °C in a humidified atmosphere of 5% CO_2_.

### Expression and purification of AG8 IgG

The pMAZ-AG8H and pMAZ-AG8L plasmids, which encode the heavy and light chains of AG8 IgG, respectively, were constructed using an eCube Plasmid DNA Mini Kit (PhileKorea, Seoul, Korea) and transfected into Expi293 cells using polyethyleneimine, as described in the literature^26^. After resuspension of the cells in 300 ml of GIBCO FreeStyle^TM^ medium (Thermo Fisher Scientific, Waltham, MA, USA), incubation at 37 °C with shaking at 125 rpm under 8% CO_2_ for 6 days, and centrifugation at 4000×*g*, the supernatants were mixed with 40 ml of 25 × PBS (pH 7.4) and 1 ml of a slurry of Protein A agarose resin (GenScript, Piscataway, NJ, USA). The resuspension was incubated at 4 °C for 16 h and passed through a polypropylene column (Thermo Fisher Scientific, Waltham, MA, USA) to recover the resin. Next, 100 ml of 1× PBS (pH 7.4) was added to the column to wash the resin, and 3 ml of 100 mM glycine-HCl buffer (pH 2.5) was loaded onto the column for elution. The eluents were immediately neutralized by the addition of 1 ml of Tris-Cl (pH 8.0). After buffer exchange with 1× PBS (pH 7.4) using Amicon Ultra^®^4 spin columns (Merck Millipore; 3-kDa cutoff), the concentration and purity of AG8 IgG were analyzed by measuring the absorbance at 280 nm and by 4–15% SDS–PAGE.

### Physicochemical analysis of AG8 IgG

Antibody aggregation was measured with a Waters Alliance 2695 system (Milford, MA, USA) and a Waters BioSuite high-resolution size-exclusion chromatography (SEC) column (7.5 mm × 300 mm, 10-μm particle size). Samples (10 μl, 1 mg/ml) were injected, and separation was conducted using isocratic elution with 1 × PBS (pH 7.4) at a flow rate of 1 ml/min. The purity was analyzed with reversed-phase high-performance liquid chromatography (RP-HPLC) using an Agilent 1260 Infinity system (Santa Clara, CA, USA). A Waters XBridge BEH 300 C4 (4.6 mm × 150 mm, 3.5-μm particle size) column was used to separate analytes at a flow rate of 1.44 ml/min. The mobile phase was 0.1% trifluoroacetic acid (TFA) in water (Eluent A) and 0.1% TFA in acetonitrile (Eluent B) applied in gradient mode: 0–18 min, a linear increase from 20 to 80% Eluent B; 18–30 min, washing, and re-equilibration. The injection concentration and volume were the same as those used for SEC. The intact masses of the antibody were determined with RP-HPLC using a Waters Acquity I class UPLC system. Separation was performed on a Thermo MabPac^TM^ RP column (2.1 mm × 50 mm, 4-μm particle size) at a flow rate of 0.2 ml/min. The mobile phase was prepared by mixing 0.1% formic acid in water (Eluent A) and 0.1% formic acid in acetonitrile (Eluent B). After linear gradient elution for 2 min with an increase in the ratio of Eluent B to 25% followed by isocratic elution with 25% Eluent B, the sample was separated by linear gradient elution (25–45% Eluent B). The effluent was analyzed with a Thermo Fisher LTQ Orbitrap mass spectrometer (Thousand Oaks, CA, USA) using Fourier transform (FT) mode. The resolution and mass range of the FT-based mass spectrometer were 120,000 and *m/z* 400–4000, respectively. The injection concentration and volume were 0.1 mg/ml and 5 μl, respectively. Glycan profiling was performed with a Rapi-Fluor labeling kit (Waters, Milford, MA, USA), and all procedures were performed as described previously^27,28^.

### ELISA

For coating, 50 μl of G_αi3_ (4 μg/ml, diluted in 0.05 M Na_2_CO_3_ (pH 9.6)) was added to a 96-well polystyrene plate, and the plate was incubated at 4 °C for 16 h. After the addition of 150 μl of 4% skim milk in 1× PBS (pH 7.4) and incubation for 2 h for blocking, 50 μl of 4 μg/ml hET_A_/mET_A_ reconstituted in 0.5% sarkosyl was added to the plate. Then, the plate was washed four times with 150 μl of 1× PBS containing 0.05% Tween 20 (PBST, pH 7.4), and 50 μl of AG8 IgG serially diluted in 1× PBS (pH 7.4) was added. After the plate was washed with 150 μl of PBST, 50 μl of a goat anti-human IgG (H + L) antibody-HRP conjugate (5000-fold dilution; Thermo Fisher Scientific, Waltham, MA, USA) was added. After the plate was washed with 150 μl of PBST, 50 μl of 1-Step™ Ultra TMB was added, the plate was incubated for 20 min, and 50 μl of 4 N H_2_SO_4_ was added to the wells to quench the ELISA signal. The absorbance at 450 nm was analyzed in an Epoch plate reader (BioTek, Winooski, VT, USA).

### Calcium flux assay

Changes in the cytosolic Ca^2+^ concentration upon hET_A_ binding to the ET-1 ligand were analyzed as described in the literature^29^. After incubation of 1 × 10^5^ hET_A_-overexpressing CHO-K1 cells or HT-29 colorectal cancer cells with 5 μM fura-2-acetoxymethyl ester (Fura-2-AM) dye at 25 °C for 1 h, serially diluted scAb AG8 was added. After incubation at 25 °C for 1 h, 10 nM ET-1 was added, and the resulting fluorescence emission at 510 nm, with separate excitation at 380 and 340 nm, was monitored using a FluoroMate FS-2 fluorescence spectrometer (Scinco, Seoul, Korea) to evaluate the Ca^2+^ concentration changes upon intracellular endothelin signaling.

### Proliferation assay

Cancer cell proliferation was analyzed using a CyQUANT^TM^ NF cell proliferation kit (Thermo Fisher Scientific, Waltham, MA, USA). Cells were seeded in 96-well plates at a density of 2–3 × 10^3^ cells/well. After 24 h of incubation, ET-1 and the anti-ET_A_ antibody were mixed at a 1:1 ratio in 2% FBS medium, and the medium was replaced with RPMI-1640 medium. After 24 h, CyQUANT^®^ NF dye reagent was added, and the cells were incubated at 37 °C for 30 min. Then, the fluorescence intensity was measured as the ratio of the fluorescence at 530 nm to that at 485 nm using an Infinite M200 Pro microplate reader (TECAN, Männedorf, Switzerland).

### Western blot analysis

Whole-cell protein lysates were prepared using RIPA buffer (iNtRON Biotechnology, Seongnam, Korea) supplemented with protease inhibitor cocktail (Roche, Basel, Switzerland), and total protein samples were quantified using a BCA Protein Assay Kit (Thermo Fisher Scientific, Waltham, MA, USA). After separation of equal amounts of the protein lysates on 10% Bis–Tris protein gels (Thermo Fisher Scientific, Waltham, MA, USA), transfer to PVDF membranes (Merck Millipore, USA), and blocking with 5% skim milk, the membranes were incubated with HRP-conjugated anti-β-actin, anti-phospho-ERK1/2, anti-total-ERK1/2, anti-phospho-AKT (S473), or anti-total-AKT antibodies (Cell Signaling Technology, Danvers, MA, USA). Then, the membranes were washed in 0.05% Tween 20 in Tris-buffered saline and incubated with a 1:5000 dilution of anti-rabbit IgG -HRP conjugate (Bio-Rad, USA) as the secondary antibody. Specific bands were detected using a WEST-ZOL plus Western Blot Detection System (iNtRON Biotechnology, Seongnam, Korea).

### RNA extraction and quantitative real-time PCR (qRT–PCR) analysis

The total RNA was isolated using an RNeasy Mini Kit (Qiagen, Germantown, MD, USA) following the manufacturer’s protocol. Reverse transcription was conducted using 1 μg of total RNA as a template and SuperScript^TM^ III Reverse Transcriptase (Thermo Fisher Scientific, Waltham, MA, USA). qRT–PCR was performed in triplicate in LightCycler^®^ 480 system with SYBR Green I Master Mix (Roche, Mannheim, Germany) and the appropriate primers (MSJ#50/MSJ#51), and the target gene expression levels were normalized to the β-actin level. The values from independent experiments were averaged, and are presented as the means ± standard deviations.

### Mouse xenograft model

The animal study was reviewed and approved by the Institutional Animal Care and Use Committee (IACUC) of the National Cancer Center Research Institute (NCCRI). The NCCRI is an Association for Assessment and Accreditation of Laboratory Animal Care International (AAALAC International)-accredited facility and abides by the Institute of Laboratory Resources (ILAR) guidelines. Five-week-old female nude mice (BALB/c nude) were purchased from OrientBio (Seongnam, Korea). After 1 week, colorectal cancer cells (2 × 10^6^) resuspended in 100 μl of 1× PBS (pH 7.4) were subcutaneously injected using a 31-gauge needle. The tumor-bearing mice were randomized into the control and treatment groups (*n* = 4 mice per group) after 7 days. Then, AG8 IgG (1.125 mg/kg) was injected intratumorally into each mouse at 2-day intervals, and 1 × PBS (pH 7.4, 50 µl/mouse) was injected as the negative control. After tumor volumes and body weights were measured prior to antibody injection, the tumors were measured using a caliper, and the volumes were calculated as follows: [*W*(width)2 × *L*(length)] × 1/2. The mice were sacrificed 27 days after cancer cell injection.

### Statistical analysis

Statistical analyses were performed with Student’s *t* test, and *P* < 0.05 was considered statistically significant.

### In silico modeling to predict the AG8 binding site in ET_A_

Structural modeling of the single-chain variable fragment antibody (scFv) was carried out using the AG8 sequence and the antibody modeling tool of the Discovery Studio 2019 program (Biovia, San Diego, CA, USA). The crystal structures for ET_B_ from the Protein Data Bank (PDB IDs: 5GLI and 5GLH for ligand-free hET_B_ and ET-1-bound ET_B_, respectively) were used for modeling and docking analysis. The potential binding site in AG8 was limited to the extracellular region of the ET_B_ structure, and the most stable binding site was determined using the “ZDOCK” function in Discovery Studio 2019^30^.

## Results

### Preparation of functional hET_A_ antigens mimicking the structure of native hET_A_ on the cell membrane

**Fig. 1:**
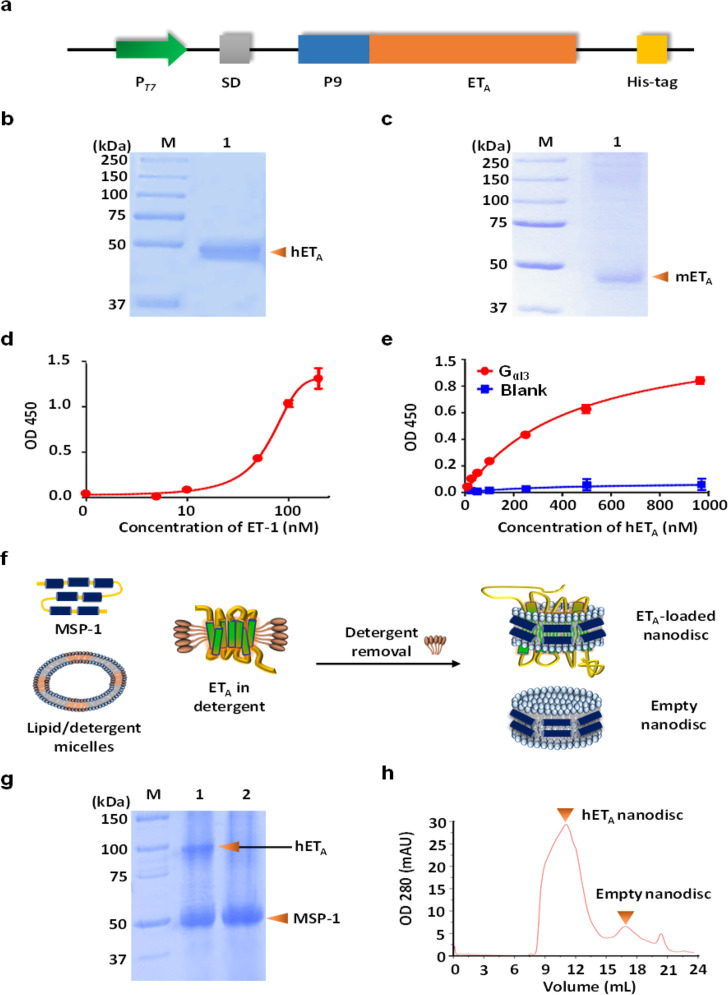
Preparation of the ET_A_ antigen for isolation of an anti-hET_A_ antibody. **a** Expression cassette for endothelin receptor type A. **b**, **c** SDS–PAGE gel images showing purified human ET_A_ (hET_A_) (**b**) and mouse ET_A_ (mET_A_) (**c**). **d**, **e** ELISA results showing the binding of purified hET_A_ to its ligands ET-1 (**d**) and G_αi3_ (**e**). **f** Overall scheme showing the method for preparing reconstituted ET_A_ nanodiscs and empty nanodiscs. **g**, **h** SDS–PAGE gel image (**g**) and gel filtration chromatogram (**h**) showing the hET_A_ nanodisc and empty nanodisc fractions; Lane 1: hET_A_ nanodisc fraction; Lane 2: empty nanodisc fraction.

For screening of monoclonal human antibodies against hET_A_, it is necessary to prepare a sufficient amount of functional antigen structurally similar to native hET_A_. However, it is well known that the complex structure of GPCRs, with seven transmembrane α-helices, is difficult to express in heterologous hosts^31^. In a previous study, we overexpressed hET_A_ in *E. coli* by fusion of the P9 sequence of *Pseudomonas* phage Ф6 (Phi6) to the N-terminal region of hET_A_ (Fig. 1a)^20^. As reported in the previous work, both hET_A_ and mET_A_ were successfully overexpressed in *E. coli* through the fusion of the N-terminal P9 motif, and they were purified from sarkosyl-solubilized cell membrane fractions using Ni-NTA affinity chromatography (Fig. 1b, c). In an ELISA, purified hET_A_ showed binding affinity not only for its ligand ET-1 but also for human G_ai3_, which is an essential component of GPCR downstream signaling^8^ (Fig. 1d, e). To prepare a functional hET_A_ antigen with a native-like structure, we reconstituted purified hET_A_, MSP-1, and lipids in an optimized ratio, and antigen-embedded nanodiscs were successfully recovered by size-exclusion chromatography (SEC) (Fig. 1f–h).

### Isolation of a human ET_A_-specific antibody using a constructed human antibody library and immobilized hET_A_ nanodiscs

**Fig. 2:**
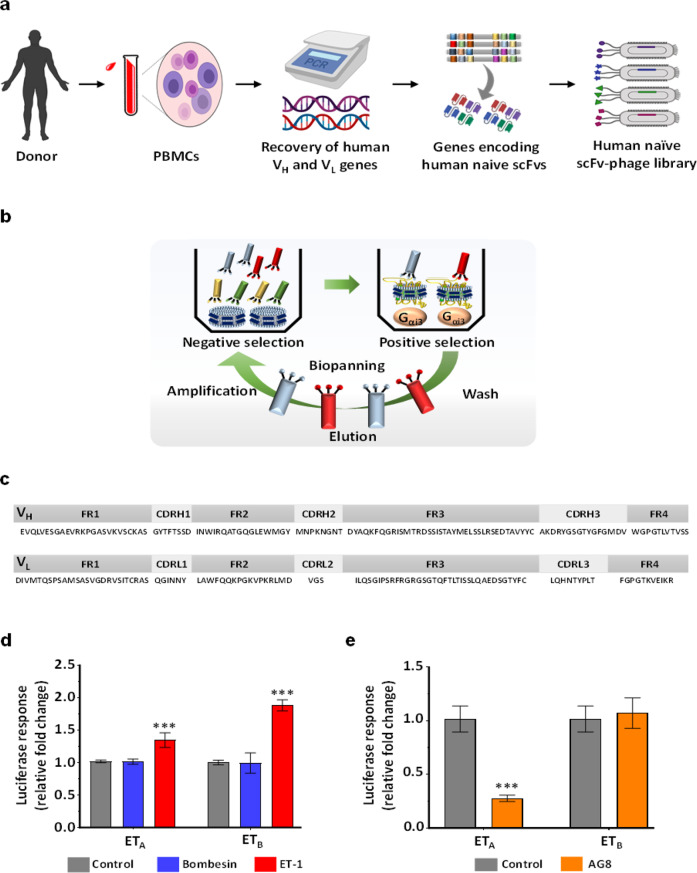
Analysis of protein sequence and endothelin receptor specificity for AG8. **a**, **b** Overall scheme showing the processes for constructing the human naïve scFv antibody library (**a**) and screening for the anti-ET_A_ antibody using the phage display system (**b**). **c** Amino acid sequences of the framework and complementarity-determining regions in AG8. **d** β-Arrestin Tango recruitment assay using bombesin and ET-1 in cells that express ET_A_/ET_B_ receptors. **e** β-Arrestin Tango recruitment assay using M13 phage particles displaying AG8 scFv. The error bars show the mean ± standard deviation values; *P ≤ 0.05, **P ≤ 0.01, ***P ≤ 0.001 vs. control.

To isolate a specific ET_A_ human antibody, we constructed a phage library displaying human scFv antibodies (library size: >1 × 10^10^ individual clones, as estimated from the number of transformants) by PCR amplification of VH and VL genes existing in the immune repertoire of human B cells (Fig. 2a). The purified hET_A_ nanodiscs were immobilized on the plate in an orientation-controlled manner through capture by precoated human G_αi3_ so that the scFv antibodies could efficiently access the extracellular region of hET_A_. After five rounds of negative screening of the phage library against empty nanodiscs and biopanning against immobilized hET_A_ nanodiscs with increasing numbers of washing cycles in successive screening rounds (Fig. 2b), we observed that phages displaying a high affinity for hET_A_ nanodiscs were enriched based on the output phage titers (Supplementary Table 3). As determined by phage ELISA, five individual clones exhibited a high signal for binding to the hET_A_ nanodisc, and DNA sequencing of the five clones revealed that all had the same scFv sequence (Fig. 2c), suggesting successful enrichment of a particular human antibody clone via the hET_A_ affinity-based screening system. Next, we aligned the sequences of the variable regions of the scFv antibody (AG8) with the germline sequences of those of human immunoglobulins using IMGT/V-QUEST^32^. The sequence analysis results revealed a sequence identity of 94.44% between the VH region of AG8 and the human immunoglobulin heavy-chain variable region V1–8 genes (IMGT ID: M99637), and the sequence of the AG8 VL light chain was 90.68% identical to that of the human immunoglobulin kappa chain variable region V1–17 genes (IMGT ID: KM455566).

### AG8 exerts antagonistic effects on ET-1-induced signaling of hET_A_

**Fig. 3:**
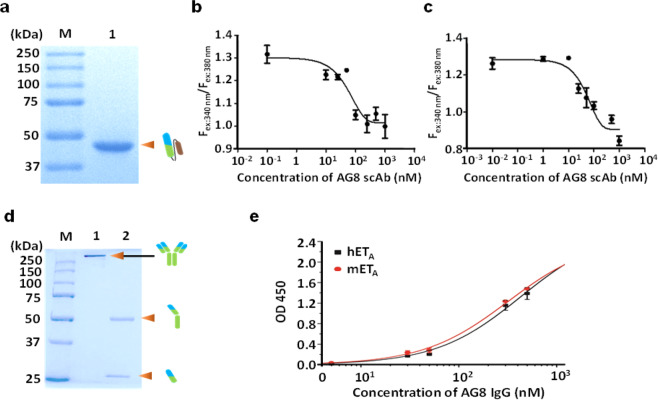
Purification and characterization of AG8. **a** SDS–PAGE gel showing the band for the purified scAb AG8. **b**, **c** Calcium flux assay with the scAb AG8 in CHO-K1 cells expressing hET_A_ (**b**) and in HT-29 cells (**c**). **d** SDS–PAGE showing purified AG8 IgG. **e** ELISA showing the cross-species binding property of AG8 IgG to human ET_A_ and mouse ET_A_.

For analysis of the antagonistic effects of the isolated antibody on ET-1-induced hET_A_ signaling, we employed a β-arrestin Tango assay that enabled monitoring of β-arrestin recruitment through luciferase gene expression^33^. In cells that expressed both hET_A_ and hET_B_, luciferase expression was not activated in the control group treated with bombesin, a ligand unrelated to both hET_A_ and hET_B_; however, the presence of ET-1, a native ligand for both hET_A_ and hET_B_, activated luciferase expression (Fig. 2d). As expected, the addition of phage particles displaying the isolated AG8 scFv inhibited luciferase expression by up to 72% in hET_A_-expressing cells. In sharp contrast, cells expressing hET_B_, which shares the capacity for ET-1 binding with hET_A_, did not exhibit a reduction in luciferase expression upon treatment with the same phage particles, indicating that the resulting AG8 is highly specific for a particular isotype of hET_A_ rather than an isotype of hET_B_ (Fig. 2e). To investigate whether purified AG8 can regulate the function of hET_A_, we expressed the isolated antibody in *E. coli* as a single-chain antibody (scAb) that contained a human kappa light-chain constant (HuCκ) domain and purified it via affinity chromatography using KappaSelect resin (Cytiva, Marlborough, MA, USA) (Fig. 3a). Then, the antagonistic effect of AG8 on hET_A_ was analyzed using fura-2-acetoxymethyl ester (fura-2 AM), a ratiometric calcium indicator, to analyze ET-1-binding-triggered hET_A_ activation, which can be monitored by measuring the increase in the intracellular Ca^2+^ level mediated through the inositol trisphosphate (IP3) pathway^34^. In both hET_A_-expressing CHO-K1 cells treated with 10 nM ET-1 and HT-29 colorectal cancer cells treated with the same concentration of ET-1, the scAb AG8 inhibited the ET-1-induced increase in the intracellular Ca^2+^ level, as evidenced by the IC_50_ values (56 nM in CHO-K1 cells and 51 nM in HT-29 cells). These results clearly demonstrate that AG8 exerted an antagonistic effect on ET-1 ligand binding-mediated hET_A_ signaling (Fig. 3b, c).

### The cross-species high binding affinity of AG8 IgG for human ET_A_ and mouse ET_A_

To verify the cross-species binding affinity of AG8 IgG for human and mouse ET_A_ antigens, AG8 was expressed in a full-length IgG form in Expi293 mammalian cells and purified via Protein A affinity chromatography (Fig. 3d). The cross-species binding of AG8 IgG was verified by ELISAs using purified human or mouse ET_A_ captured by human G_αi3_ that was preimmobilized on ELISA plates. Considering that the protein sequence of the mouse ET_A_ antigen is 94.3% identical to that of human ET_A_, we reasoned that AG8 IgG would show a binding affinity for both hET_A_ and mET_A_ proteins. As expected, the apparent binding affinities of AG8 IgG for human and mouse ET_A_ were almost identical in the ELISAs (Fig. 3e).

### Physicochemical properties of AG8 IgG

**Fig. 4:**
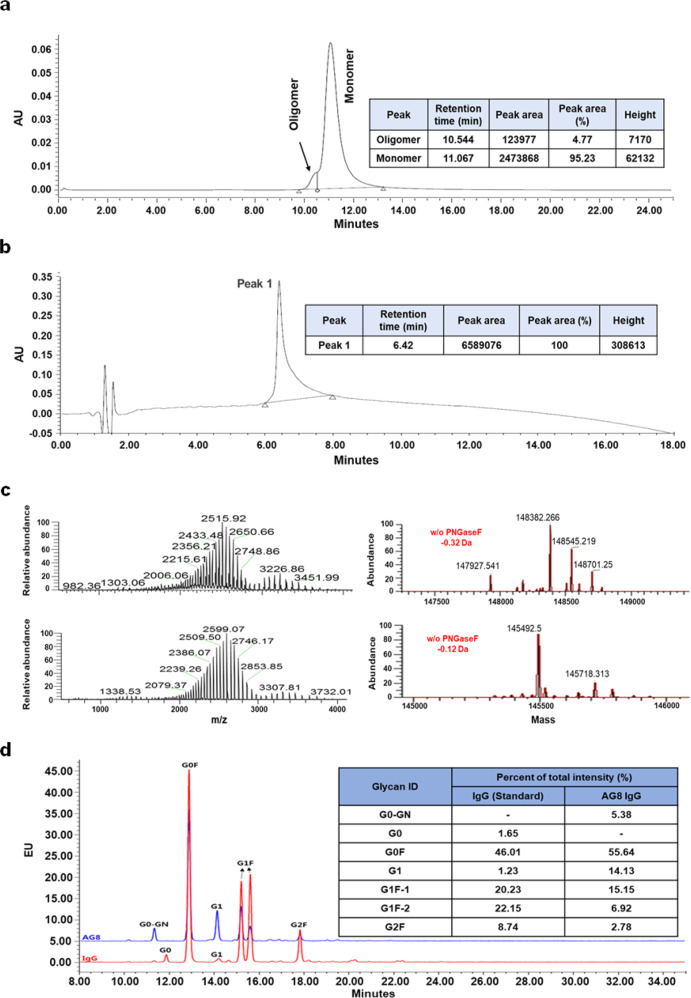
Physicochemical characterization of AG8. **a** SEC analysis of the oligomer proportions of purified AG8 IgG. **b** RP-HPLC chromatogram generated to analyze the purity of AG8 IgG. **c** Intact mass analysis for purified AG8 IgG. **d** HPLC analysis for glycan profiling of AG8 IgG.

The physicochemical properties of AG8 IgG were characterized by four methods, as shown in Fig. 4. The percentages of the monomeric and aggregated forms of AG8 IgG were 95.23% and 4.77%, respectively, and no other impurities were detected in RP-HPLC analysis. The molecular weight of AG8 IgG was measured both with and without PNGase F treatment, and the accuracies were < 1 Da. The glycan profile of AG8 IgG was slightly different from that of the IgG standard, but the result was similar to those shown in other reports^27,28^. No analyzed physicochemical properties created an issue for subsequent in vitro and in vivo assays.

### In vitro effects of AG8 IgG on cancer cells

**Fig. 5:**
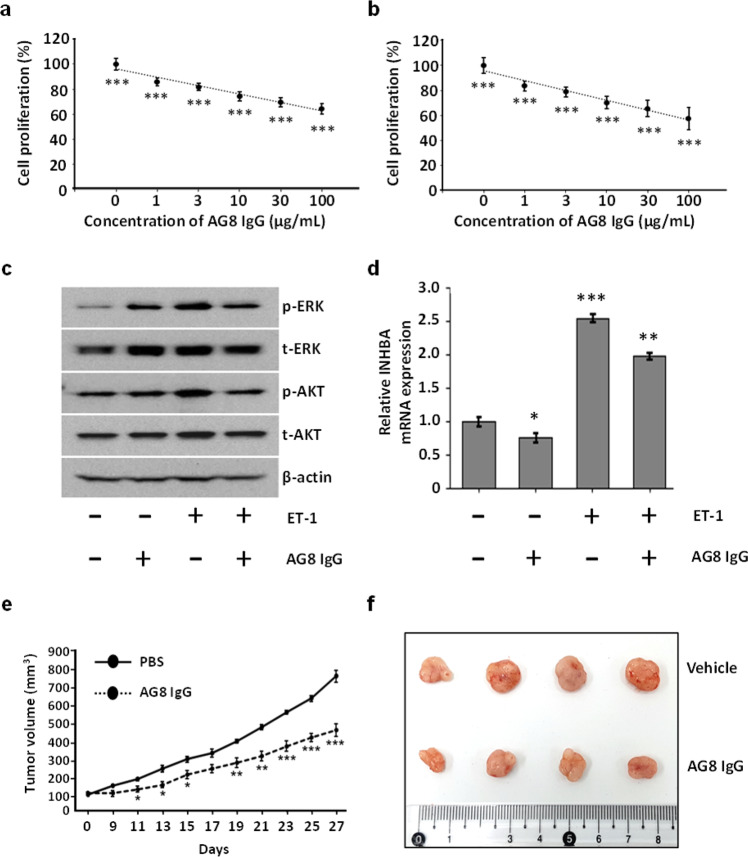
Anti-hET_A_ AG8 suppressed the growth of colorectal cancer cells. **a**, **b** Inhibition of the proliferation of colorectal cancer cells (HT-29 (**a**) and HCT-116 (**b**)) treated with AG8 IgG. Cancer cells were seeded in 96-well plates in the presence of AG8 and incubated for 72 h. **c** Western blot analysis of phosphorylated AKT and ERK levels in HCT-116 colorectal cancer cells treated with ET-1 (10 nM) for 10 min with or without pretreatment with AG8 (100 μg) for 4 h. β-Actin was used as the loading control. **d** The mRNA expression of INHBA in HCT-116 cells treated with ET-1 (10 nM) for 24 h with or without pretreatment with AG8 (100 μg) for 4 h. **e** HT-29 cells were subcutaneously injected into nude mice. Tumor-bearing mice were randomized, and AG8 (1.125 mg/kg) was intratumorally injected into each mouse at intervals of 2 days. **f** Photos of dissected tumor masses on Day 27. The error bars show the mean ± standard deviation values; *P ≤ 0.05, **P ≤ 0.01, ***P ≤ 0.001 vs. control.

Human ET_A_ is an important target for cancer treatment because it is highly involved in several signaling pathways that promote cell proliferation, metastasis, and neovascularization^34^. In particular, a high correlation between hET_A_ overexpression and the progression of colorectal cancer has been reported^35^. In two colorectal cancer cell lines, HT-29 and HCT-116, AG8 IgG reduced the proliferation of cells by up to 40% (Figs. 5a and 5b). To investigate how AG8 IgG inhibited the proliferation of these cells, we performed western blot analyses to measure the phosphorylation levels of downstream signaling pathway components. It has been well established that ET-1 binding to hET_A_ promotes the phosphorylation of ERK and AKT in colorectal cancer cells^36,37^. We found that the addition of AG8 IgG significantly reduced ET-1-induced phosphorylation of both ERK and AKT in cancer cells (Fig. 5c). Furthermore, transcription of inhibin βA (INHBA), which is activated by ET-1 binding to hET_A_, was decreased upon treatment with AG8 IgG (Fig. 5d). Taken together, these results indicate that the specific binding of AG8 IgG to hET_A_ blocks downstream hET_A_ signaling and inhibits colorectal cancer cell proliferation.

### Inhibition of tumor growth by AG8 IgG in BALB/c nude mice

We next confirmed the anticancer effects of AG8 IgG in vivo. A xenograft mouse model was established by subcutaneous injection of colorectal cancer cells into the flanks of BALB/c nude mice, and AG8 IgG was administered at 2-day intervals by intratumoral injection (1.125 mg/kg per injection). After 27 days, tumor growth in the AG8-treated mice was decreased 40% relative to that in PBS-treated mice (Fig. 5e, f), clearly showing that AG8 IgG exerted significant antitumor effects in mice bearing colorectal cancer xenografts.

### In silico modeling of the structure of AG8

**Fig. 6:**
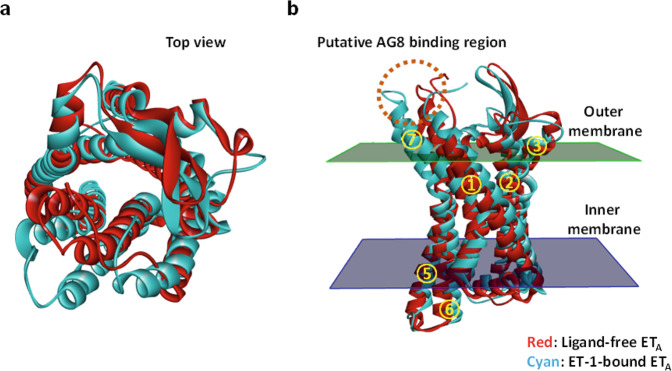
Conformational change in the hET_A_ model structure induced by ET-1 binding. **a** Structures of ligand-free ET_A_ (red) and ET-1-bound ET_A_ (cyan). The structures are viewed from the outside of the cell (top view). **b** The structure as viewed from the side. The binding site of AG8 predicted by protein docking analysis is represented by a dotted circle. The numbers 1 through 7 in the yellow circles indicate the transmembrane domain of hET_A_.

Comparison of the sequences of the hET_A_ (UniProtKB ID: P25101) and hET_B_ (UniProtKB ID: P24530) proteins determined their sequence identity and similarity to be 53.9% and 71.8%, respectively. To infer the structure of hET_A_, for which no crystal structure is available, two crystal structures (PDB codes: 5GLI and 5GLH for ligand-free hET_B_ and ET-1-bound ET_B_, respectively) were used for in silico analysis. Models of both ligand-free and ligand-bound hET_A_ were constructed using the sequence of hET_A_ and the two crystal structures of hET_B_ (5GLI and 5GLH). Superimposition of the resulting two hET_A_ models showed that the root-mean-square deviation between the two models was 2.694 Å (Fig. 6a). The in silico analysis showed that the endothelin-binding site in hET_A_ was located in the region inside the 7 transmembrane helices, as in the ET-1-bound ET_B_ structure^38^, and that the conformations of two transmembrane helices (TM6 and TM7) were changed more significantly than those of the other transmembrane helices upon binding to ET-1. To analyze the AG8 binding site in hET_A_, a structural model of AG8 was generated using Discovery Studio 2019, and the potential binding sites were listed in order of stabilization energy using the docking function of the software. The results revealed that the extracellular loop 3 (ECL3) region connected to the 6th and 7th transmembrane helices of hET_A_ showed the most stable binding (Fig. 6b). Interestingly, this region exhibited the highest degree of conformational change upon binding to ET-1.

## Discussion

In this study, we overexpressed a type of GPCR with an intrinsically complex structure using a bacterial expression system and prepared a protein in the form of a nanodisc to maintain a GPCR structure similar to that of native GPCRs expressed in the cell membrane environment^22^. This antigen preparation strategy enabled us to isolate a human anti-GPCR antibody with high target antigen selectivity and the capability to regulate intrinsic GPCR function. Although various GPCR antigen preparation methods, such as fusion of the GPCR extracellular region with a carrier protein, production of membrane fractions containing GPCR proteins, and synthesis of peptides of GPCR extracellular regions, have been used for GPCR antibody screens, these methods have limitations due to their strong tendency to generate GPCR conformations different from those of the native GPCR expressed in the human cell membrane, the low stability of the GPCR antigen during antibody screening steps, and the occurrence of structural modifications during chemical conjugation or genetic fusion of a part of a GPCR antigen with a carrier protein^17^. Our group has also fused carrier proteins such as keyhole limpet hemocyanin and ovalbumin with synthetic peptides encoding the N-terminus, extracellular loop 1 (ECL1), extracellular loop 2 (ECL2), or extracellular loop 3 (ECL3) of hET_A_ for isolation of anti-GPCR antibodies. However, our antibody screening trial performed through animal immunization using the prepared antigen consisting of a synthetic GPCR peptide subunit fused with a carrier protein was not successful. To overcome these limitations, we prepared a GPCR antigen in nanodisc form. Nanodiscs reconstituted with a protein such as a GPCR, phospholipids, and MSPs have been used in various studies on membrane proteins^17,39^. Cai et al. solubilized the human glucagon-like peptide-1 receptor (GLP-1R) with detergent and successfully produced a nanodisc using MSP and phospholipids, and they confirmed binding activity with its ligand GLP-1 and with the G_S_ protein^40^. In a similar way, the self-assembly of detergent-solubilized hET_A_ with POPC and MSP enabled us to produce hET_A_ nanodiscs_,_ leading to successful isolation of an hET_A_-specific human antibody.

Through a β-arrestin recruitment Tango assay^33^, we confirmed that the isolated antibody AG8 selectively bound to hET_A_, enabling the regulation of downstream hET_A_ signaling. Aberrant activation and overexpression of hET_A_ have an important effect on the survival of patients with a variety of cancers, such as breast, cervical, colorectal, ovarian, prostate, and head and neck cancers^14,41^. Currently, the main antagonists targeting endothelin receptors approved for clinical trials include sitaxentan, bosentan, macitentan, and ambrisentan, all of which are small-molecule compounds. A clinical trial for sitaxentan was withdrawn, and bosentan and macitentan are dual ET_A_ and ET_B_ antagonists, whereas ambrisentan is the only antagonist known to selectively bind to ET_A_^42^. Kappes et al. used ambrisentan in a preclinical murine model of metastatic breast cancer and confirmed that it inhibited cancer cell migration, invasion, and metastasis by selectively binding to ET_A_ without interfering with the physiological vasodilator function controlled by ET_B_^43^. This suggests that selective binding of antagonists to a specific type of endothelin receptor is likely to be beneficial for cancer therapy. AG8 IgG, with high ET_A_ selectivity, could be a candidate therapeutic agent for cancers in which patient survival prognosis is affected by dysfunction or overexpression of ET_A_.

The protein sequence of hET_A_ is 94% identical to that of its mouse homolog^6^. As expected, AG8 IgG showed cross-reactivity with both human and mouse ET_A_. In the development of anticancer therapeutic antibodies, it is necessary to evaluate antitumor effects using small animal models such as mouse models prior to assessing efficacy in primates and humans. If the antibody binds to the human antigen but not to the corresponding antigen expressed in the animal model, a surrogate antibody with characteristics and binding properties similar to those of the counterpart antigen in the model animal should be produced. Alternatively, a knock-in animal model expressing the human target antigen should be used. As mentioned above, ET_A_ has high sequence identity between humans and mice. In addition, the sequences of its ligands ET-1 and ET-3 are identical between the two species, and another endothelin ligand, ET-2, exhibits substantial similarity (95.2%) between humans and mice, suggesting that it is reasonable to evaluate the antitumor effects of AG8 IgG in a non-transgenic mouse xenograft model.

Representative therapeutic antibodies used for colorectal cancer treatment are bevacizumab and cetuximab. These two drugs have been administered in combination with small-molecule drugs such as irinotecan, oxaliplatin, and fluoropyrimidines in treatment regimens^44,45^. However, long-term treatment with bevacizumab usually increases the expression of soluble VEGF receptor 1 (sVEGFR1) and results in resistance to the drug^46,47^. In addition, cetuximab shows a general loss of therapeutic efficacy in patients with K-RAS mutations^48^. Therefore, there is an urgent unmet clinical need for the development of improved therapeutic agents for colorectal cancer. ET_A_ is activated by both the paracrine and autocrine systems; thus, it affects cancer progression and metastasis in a variety of ways^12,14,49–53^. In this study, we conducted in silico analysis, and the results showed that the ET_A_ mRNA expression level in colorectal cancer was higher than that in other cancers (breast, cervical, ovarian, prostate, and head and neck cancers) (Supplementary Fig. 1). Currently, small-molecule-based ET_A_ antagonists with FDA approval for treating hypertension, kidney diseases, and heart failure have been reported to inhibit tumor progression in a variety of cancers^54–56^. The IgG antibody AG8 isolated in this study inhibited cell growth by specifically binding to ET_A_ in colorectal cancer cells, increasing the cytosolic Ca^2+^ level and blocking the activation of ET_A_ downstream signaling. In addition, the antitumor efficacy of AG8 IgG was confirmed in a colorectal cancer xenograft model. The tumor growth inhibition observed here was superior to that in a previous study based on a single administration of bevacizumab^57^. These results can be explained by the decreases in the levels of phosphorylated AKT, phosphorylated ERK, and cytosolic Ca^2+^, as well as the transcription of the colorectal cancer biomarker INHBA^58^, which led to inhibition of cancer cell proliferation and growth. Therefore, the results of these AG8 IgG analyses indicate that this antibody has a mechanism of action different from those of conventional agents for the treatment of patients with colorectal cancer that is resistant to bevacizumab and cetuximab.

To identify the specific binding epitope on hET_A_ recognized by AG8 IgG, mass spectrometry (MS) analysis using hydrogen-deuterium exchange (HDX), surface plasmon resonance analysis, and ELISA using individual hET_A_ extracellular loop (ECL) peptides were conducted. However, the HDX-MS analysis was not successful due to the instability of detergent-solubilized hET_A_ during sample preparation. In addition, AG8 IgG did not bind to each individual ECL domain of hET_A_, implying that each domain of the prepared hET_A_ had a conformation different from that in the native full-length hET_A_ protein. However, we cannot exclude the possibility that multiple domains in addition to one N-terminal region or ECL loop are involved in binding to AG8 IgG. To solve the tertiary structure of hET_A_ and elucidate the interaction between hET_A_ and AG8 IgG, we plan to replace the third intracellular loop (ICL3) with T4 lysozyme to facilitate crystallization by stabilizing the conformation of ICL3, the most flexible region in a GPCR, as reported in a previous study^59^.

In this study, a highly challenging GPCR antigen was prepared, and a human antibody that selectively binds to hET_A_ was successfully isolated from a human antibody library using a phage display technique. The resulting AG8 IgG showed potent antitumor effects against colorectal cancer. To develop an antitumor therapeutic antibody with enhanced efficacy, we have engineered framework regions of the variable regions of AG8 IgG and isolated new antibodies exhibiting higher binding affinity than the parental AG8 IgG for hET_A_. Furthermore, we will validate the antitumor efficacy and pharmacokinetics of the antibody by introducing an engineered Fc variant with a prolonged circulating half-life or enhanced effector functions. In addition, the search for additional applicable therapeutic indications is in progress, and the results will be reported in our future publications.

## Supplementary information

Supplementary Information
