# Supplementary material for: A human antibody against human endothelin receptor type A that exhibits antitumor potency
Source: Exp Mol Med. 2021 Sep 29;53(9):1437–48. doi: 10.1038/s12276-021-00678-9 (PMC8492878; doi:10.1038/s12276-021-00678-9)
Supplement: Supplementary file 1 — Supplementary Information [file 12276_2021_678_MOESM1_ESM.pdf]

SUPPLEMENTARY INFORMATION

A Human Antibody against Human Endothelin Receptor Type A that Exhibits Antitumor Potency

Man-Seok Ju[1,2,#], Hye-Mi Ahn[3,#], Seong-Gu Han[4,#], Sanghwan Ko[1,2], Jung-Hyun Na[5,6], Migyeong Jo[1,7], Chung Su Lim[8], Byoung Joon Ko[9], Yeon Gyu Yu[4], Won-Kyu Lee[8,*], Youn-Jae Kim[3,*], Sang Taek Jung[1,2,7,*]

[1]*Department of Biomedical Sciences, Graduate School, Korea University, Seoul, 02841, Republic of Korea*

[2]*Institute of Human Genetics, Korea University College of Medicine, Seoul, 02841, Republic of Korea*

[3]*Division of Translational Science, Research Institute, National Cancer Center, Goyang, Gyeonggi-do, 10408, Republic of Korea*

[4]*Biopharmaceutical Chemistry Major, School of Applied Chemistry, Kookmin University, Seoul, 02707, Republic of Korea*

[5]*Department of Pharmaceutical Engineering, Sangji University, Wonju-si, Gangwon-do, 26339, Republic of Korea*

[6]*Research Institute of Korean Medicine, Sangji University, Wonju-si, Gangwon-do, 26339, Republic of Korea*

[7]*BK21 Graduate Program, Department of Biomedical Sciences, Korea University College of Medicine, Seoul, 02841, Republic of Korea*

[8]*New Drug Development Center, Osong Medical Innovation Foundation, Cheongju,*

Chungcheongbuk-do, 28160, Republic of Korea

[9]*School of Biopharmaceutical and Medical Sciences, Sungshin Women's University, Seoul, 02844, Republic of Korea*

[#]These authors contributed equally to this work.

*__Correspondence__:

Sang Taek Jung (Email: sjung@korea.ac.kr)

Youn-Jae Kim (Email: yjkim@ncc.re.kr)

Won-Kyu Lee (Email: gre7@kbiohealth.kr)

Supplementary Figure Legends

Supplementary Figure 1. Analysis of ET_A mRNA expression in normal colon and tumor colon tissues from the Oncomine database (n is number of samples).

Supplementary Table 1. Primers used in this study (underlining indicates the restriction enzyme sites).

Supplementary Table 2. Plasmids used in this study.

Supplementary Table 3. Output phage titers after each round of screening of the human scFv phage display library against human ET_A.

Supplementary Figure 1. Analysis of ET_A mRNA expression in normal colon and tumor colon tissues from the Oncomine database (n is number of samples).

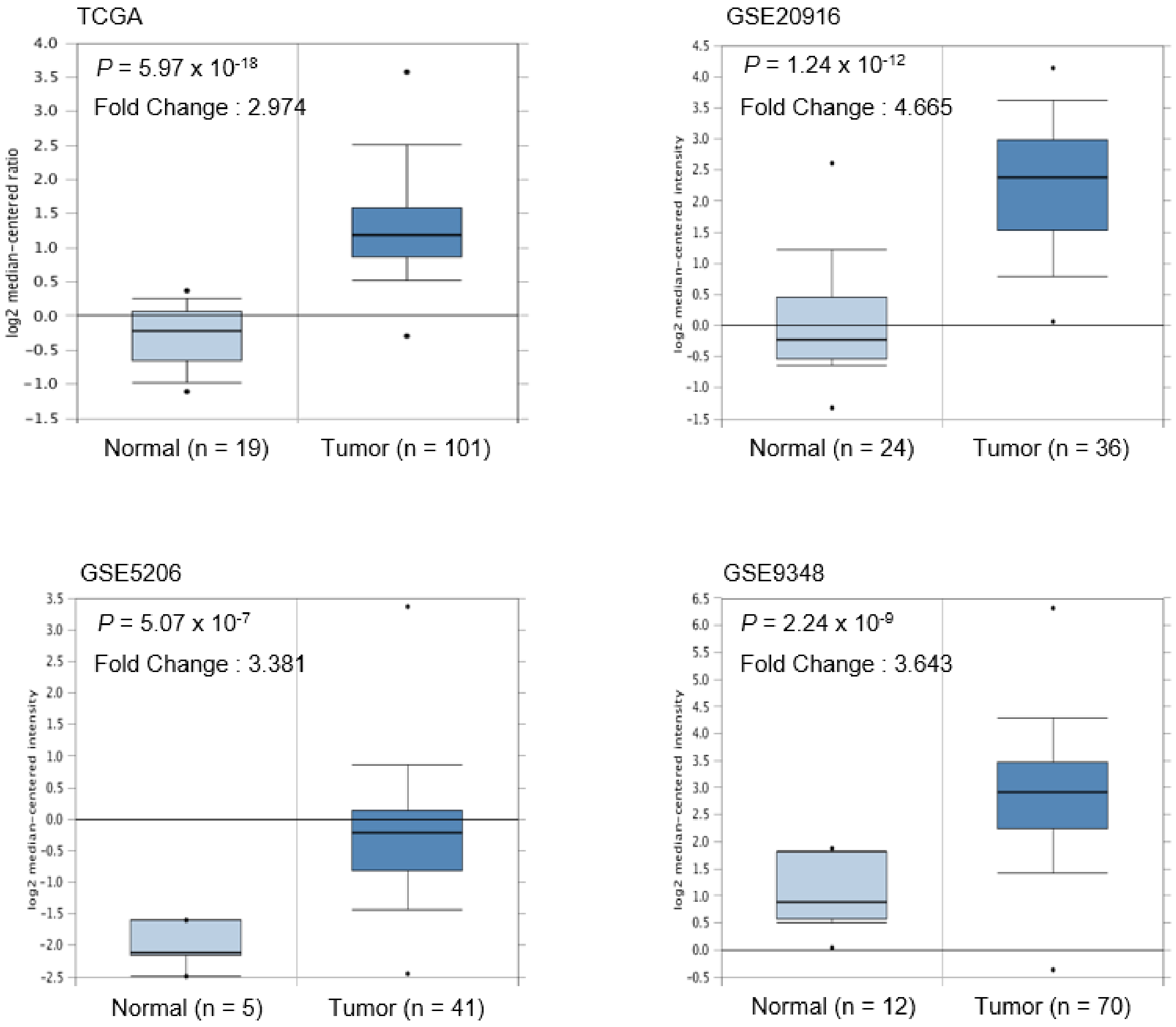

Supplementary Table 1. Primers used in this study (underlining indicates the restriction enzyme sites).

Primer Name	Primer Nucleotide Sequence (5’ → 3’)
MSJ#01	GAGCCTGGTGCCGCGCGGCTCCCGGGACAACCCTGAGAGATACAGTGCAA
MSJ#02	GGTGATGGTGATGGTGAGAAGCTTCATTCATGGAGTCTTTATGCGACGAA
MSJ#03	GCGGAATTC<u>CATATG</u>CTAAAGCTCCTTGACAACTGGGACAGCGTGACCTCCACCTTCAGC<br>AAGCTGCGCGAACAGCTCGGCCCTGTGACCCAGGAGTTCTGGGATAACCTGGAAAAGG
MSJ#04	CTCCTGCCACTTCTTCTGGAAGTCGTCCAGGTAGGGCTGCACCTTGGCCTTCACCTCCT<br>CCAGATCCTTGCTCATCTCCTGCCTCAGGCCCTCTGTCTCCTTTTCCAGGTTATCCCAGAA
MSJ#05	CCAGAAGAAGTGGCAGGAGGAGATGGAGCTCTACCGCCAGAAGGTGGAGCCGCTGCGCGC<br>AGAGCTCCAAGAGGGCGCGCGCCAGAAGCTGCACGAGCTGCAAGAGAAGCTGAGCCCACT
MSJ#06	GGCGCGCGGCCAAGCGCTGGCGCAGCTCGTCGCTGTAGGGGGCCAGATGCGTGCGCAGC<br>GCGTCCACATGGGCGCGCGCGCGGTCGCGCATCTCCTCGCCCAGTGGGCTCAGCTTCTCTT
MSJ#07	TTGGCCGCGCGCCTTGAGGCTCTCAAGGAGAACGGCGGCGCCAGACTGGCCGAGTACCAC<br>GCCAAGGCCACCGAGCATCTGAGCACGCTCAGCGAGAAGGCCAAGCCCGCGCTCGAGGAC
MSJ#08	GAATTC<u>GGATCC</u>TCACTGGGTGTTGAGCTTCTTAGTGTACTCCTCGAGAGCGCTCAGGAAG<br>CTGACCTTGAAGCTCTCCAGCACGGGCAGCAGGCCTTGGCGGAGGTCCTCGAGCGCGGG
MSJ#09	GCTACCGT<u>GGCCCAGCCGGCC</u>ATGGCGATGGCGCAGGTGCAGCTGGTGCAGTCTGG
MSJ#10	GCTACCGT<u>GGCCCAGCCGGCC</u>ATGGCGATGGCGCAGGTCAACTTAAGGGAGTCTGG
MSJ#11	GCTACCGT<u>GGCCCAGCCGGCC</u>ATGGCGATGGCGGAGGTGCAGCTGGTGGAGTCTGG
MSJ#12	GCTACCGT<u>GGCCCAGCCGGCC</u>ATGGCGATGGCGCAGGTGCAGCTGCAGGAGTCGGG
MSJ#13	GCTACCGT<u>GGCCCAGCCGGCC</u>ATGGCGATGGCGGAGGTGCAGCTGTTGCAGTCTGC
MSJ#14	GCTACCGT<u>GGCCCAGCCGGCC</u>ATGGCGATGGCGCAGGTACAGCTGCAGCAGTCAGG
MSJ#15	CAGATCCACCTCCACCGGAACCTCCACCTCCAGAGCTCCCTCCACCTGAGGAGACGGTGACCAGGGTGCC
MSJ#16	CAGATCCACCTCCACCGGAACCTCCACCTCCAGAGCTCCCTCCACCTGAAGAGACGGTGACCATTGTCCC
MSJ#17	CAGATCCACCTCCACCGGAACCTCCACCTCCAGAGCTCCCTCCACCTGAGGAGACGGTGACCAGGGTTCC
MSJ#18	CAGATCCACCTCCACCGGAACCTCCACCTCCAGAGCTCCCTCCACCTGAGGAGACGGTGACCGTGGTTCC
MSJ#19	GGTTCCGGTGGAGGTGGATCTGGTGGAGGTGCTAGCGACATCCAGATGACCCAGTCTCC
MSJ#20	GGTTCCGGTGGAGGTGGATCTGGTGGAGGTGCTAGCGATGTTGTGATGACTCAGTCTCC
MSJ#21	GGTTCCGGTGGAGGTGGATCTGGTGGAGGTGCTAGCGAAATTGTGTTGACGCAGTCTCC
MSJ#22	GGTTCCGGTGGAGGTGGATCTGGTGGAGGTGCTAGCGACATCGTGATGACCCAGTCTCC
MSJ#23	GGTTCCGGTGGAGGTGGATCTGGTGGAGGTGCTAGCGAAACGACACTCACGCAGTCTCC
MSJ#24	GGTTCCGGTGGAGGTGGATCTGGTGGAGGTGCTAGCGAAATTGTGCTGACTCAGTCTCC
MSJ#25	GGTTCCGGTGGAGGTGGATCTGGTGGAGGTGCTAGCCAGTCTGTGTTGACGCAGCCGCC
MSJ#26	GGTTCCGGTGGAGGTGGATCTGGTGGAGGTGCTAGCCAGTCTGCCCTGACTCAGCCTGC
MSJ#27	GGTTCCGGTGGAGGTGGATCTGGTGGAGGTGCTAGCTCCTATGTGCTGACTCAGCCACC
MSJ#28	GGTTCCGGTGGAGGTGGATCTGGTGGAGGTGCTAGCTCTTCTGAGCTGACTCAGGACCC
MSJ#29	GGTTCCGGTGGAGGTGGATCTGGTGGAGGTGCTAGCCACGTTATACTGACTCAACCGCC
MSJ#30	GGTTCCGGTGGAGGTGGATCTGGTGGAGGTGCTAGCCAGGCTGTGCTCACTCAGCCGTC
MSJ#31	GGTTCCGGTGGAGGTGGATCTGGTGGAGGTGCTAGCAATTTTATGCTGACTCAGCCCCA
MSJ#32	GGTGCT<u>GGCCCCCGAGGCC</u>CGTACGTTTGATTTCCACCTTGGTCCC
MSJ#33	GGTGCT<u>GGCCCCCGAGGCC</u>CGTACGTTTGATCTCCAGCTTGGTCCC
MSJ#34	GGTGCT<u>GGCCCCCGAGGCC</u>CGTACGTTTGATATCCACTTTGGTCCC
MSJ#35	GGTGCT<u>GGCCCCCGAGGCC</u>CGTACGTTTGATCTCCACCTTGGTCCC
MSJ#36	GGTGCT<u>GGCCCCCGAGGCC</u>CGTACGTTTAATCTCCAGTCGTGTCCC
MSJ#37	GGTGCT<u>GGCCCCCGAGGCC</u>CGTACCTAGGACGGTGACCTTGGTCCC
MSJ#38	GGTGCT<u>GGCCCCCGAGGCC</u>CGTACCTAGGACGGTCAGCTTGGTCCC
MSJ#39	GGTGCT<u>GGCCCCCGAGGCC</u>CGTACCTAAAACGGTGAGCTGGGTCCC
MSJ#40	GCTACCGT<u>GGCCCAGCCGGCC</u>ATGGCG
MSJ#41	<u>GGCCTCGGGGGCC</u>AGCACC
MSJ#42	CCACAG<u>GCGCGC</u>ACTCCGAGGTGCAGCTGGTGGAG
MSJ#43	CCTGGTCACCGTCTCCTCAGCAAGCTTCAAGGGCCCA
MSJ#44	TGGGCCCTTGAAGCTTGCTGAGGAGACGGTGACCAGG
MSJ#45	CGAGCT<u>TCTAGA</u>TCATTTACCCGGGGACAGGGAG
MSJ#46	CCACAG<u>GCGCGC</u>ACTCCGGACATCGTGATGACCCAGTCTCC
MSJ#47	GGACCAAGGTGGAGATCAAACGTACGGCGGCCGCAC
MSJ#48	GTGCGGCCGCCGTACGTTTGATCTCCACCTTGGTCC
MSJ#49	CGAGCT<u>TCTAGA</u>GCACTCTCCCCTGTTGAAGC
MSJ#50	GGTTCCCTTGTGAGCCTTGA
MSJ#51	AGGAAASGTGCCTGTGATGGG

Supplementary Table 2. Plasmids used in this study.

Plasmid	Relevant characteristics	Reference or source
pP9	Ap_r, T7 promoter, thrombin cleavage site, P9 gene in $pRSET_A$	[20]
pP9-mET_A	Ap_r, T7 promoter, thrombin cleavage site,<br>P9 gene, mouse ET_A in pP9	This study
pP9-hET_A	Ap_r, T7 promoter, thrombin cleavage site,<br>P9 gene, human ET_A in pP9	[20]
pET28a(+)	Kan_r, T7 promoter, thrombin cleavage site, polyhistidine tag	Novagen
pET28-MSP1	Kan_r, T7 promoter, thrombin cleavage site,<br>N-terminal polyhistidine tag	This study
pMAZ-IgH-GlycoT	Trastuzumab H chain gene in pMAZ-IgH-H23	[21]
pMAZ-IgL-GlycoT	Trastuzumab L chain gene in pMAZ-IgL-H23	[21]
pComb3X	Ap_r, lac promoter, polyhistidine tag, HA tag in phagemid	[24]
pEL3X	Modified *Sfi*I restriction nucleotide sequence in pComb3X	This study
pET28a-$G_{\alpha i3}$	Kan_r, T7 promoter, thrombin cleavage site,<br>N-terminal polyhistidine tag, human $G_{\alpha i3}$ gene in pET28a(+)	[25]

Supplementary Table 3. Output phage titers after each round of screening of the human scFv phage display library against human ET_A.

Round	1	2	3	4	5
Number of washes	2	3	3	6	10
Output phage titer (cfu/ml)	2×10^6	1.5×10^6	2×10^6	1.8×10^5	1×10^6
